# A Markov cost‐effective analysis of biannual fluoride varnish for preventing dental caries in permanent teeth over a 70‐year time horizon

**DOI:** 10.1002/hpja.283

**Published:** 2019-08-27

**Authors:** Tan Minh Nguyen, Utsana Tonmukayakul, Emma Warren, Susan Cartwright, Danny Liew

**Affiliations:** ^1^ Deakin Health Economics, Institute of Health Transformation, Faculty of Health Deakin University Waurn Ponds Vic. Australia; ^2^ Melbourne Dental School The University of Melbourne Melbourne Vic. Australia; ^3^ Hera Consulting Balmain NSW Australia; ^4^ Colgate‐Palmolive Company Sydney NSW Australia; ^5^ Nursing and Health Sciences, School of Public Health & Preventive Medicine, Faculty of Medicine Monash University Clayton Vic. Australia

**Keywords:** cost‐benefit analysis, dental care, dental caries, fluorides, primary health care

## Abstract

**Issue addressed:**

Biannual application of fluoride varnish is effective for dental caries prevention, but its cost‐effectiveness using quality‐adjusted life years (QALY) is unknown. This study performed a cost‐effectiveness analysis, from the Australian health care system perspective of biannual application of fluoride varnish versus current practice (non‐routine application) for an individual aged 15 years and older over a 70‐year time horizon.

**Methods:**

Health outcomes measured were the number of prevented decayed, missing, and filled teeth (prevented‐DMFT) and QALY gained. The calculated incremental cost‐effectiveness ratio (ICER) was compared against the reference cost‐effectiveness ICER threshold of AUD$28 033 per QALY gained. A published Markov model capturing dental caries progression of eight permanent molars was used. This 6‐monthly cycle model represented ten possible health states for an individual tooth. A 5% discount rate was applied with relevant sensitivity analysis.

**Results:**

In the base‐case scenario, the net cost for the intervention was $3600 compared to $2303 in the current practice arm. The intervention arm yielded 13.99 DMFT and 15.44 QALY gained, whereas the current practice arm yielded 15.52 DMFT and 14.74 QALY gained. The estimated ICER was $849 per prevented‐DMFT and $1851 per QALY gained. Sensitivity analysis shows the ICER ranged from $424‐$1807 per prevented‐DMFT and $1851‐$3941 per QALY gained.

**Conclusion:**

Biannual professional application of fluoride varnish appears to be a highly cost‐effective strategy and should be considered for universal funding in Australia's health care system.

## Summary

Biannual application of fluoride varnish is efficacious at preventing dental caries among children, but its cost‐effectiveness in terms of health outcomes is unknown. This study performed a cost‐effectiveness analysis, from the perspective of the Australian health care system. The study demonstrated the intervention is likely to be highly cost‐effective.

## 1 INTRODUCTION

Dental caries in permanent teeth is highly prevalent among Australian children. By age 12‐14 years, approximately 38% of children will have had some level of caries experience; 15% have untreated dental caries.^1^ The cost to surgically manage dental caries is expensive and ranges from simple restorations to more complex procedures, including crowns, root canal treatment and tooth extractions, which could lead to tooth replacement prosthetics such as dental implants, bridge and dentures.

Biannual application of fluoride varnish to teeth can prevent dental caries development and progression. Fluoride varnish enhances the remineralisation process of early caries lesions in combination with calcium and phosphate ions, resulting in mineral formation that makes enamel and dentine more resistant to acid challenge.^2^ A Cochrane systematic review and meta‐analysis found that fluoride varnish has caries inhibiting efficacy in both permanent and primary teeth of children and adolescents, compared to no treatment.^3^ This finding was supported by a more recent review that found benefits for the intervention across all ages.^4^

Despite established efficacy, professionally applied fluoride varnish has not had widespread practice.^5^, ^6^ In countries with community water fluoridation such as Australia, the Republic of Ireland and the US, fluoride varnish is recommended for elevated caries risk, whereas in countries with limited or no water fluoridation such as England and Scotland, fluoride varnish is recommended for all children and young adults.^7^ The Australian fluoride guidelines state that “fluoride varnish should be used for people who have elevated risk of developing caries.”^8^

Fluoride varnish is funded under the Child Dental Benefits Scheme (CDBS) for eligible children aged between 2 and 17 years, state/territory public dental services, and in part through the subsidised private health insurance rebate scheme. The CDBS is a federal dental program that provides up to AUD$1000 worth of dental care over 2 years. In Europe, Scandinavia and Canada, professionally applied fluoride varnish is publicly funded for susceptible individuals.^3^ In the US, most states reimburse applications of fluoride varnish provided by primary health care medical providers for young children in addition to those provided in dental settings.^9^

Australian‐based economic evaluations of preventive oral health interventions are limited.^10^, ^11^ Most report using the common dental caries outcome measure: the decayed, missing and filled teeth (DMFT) index.^10^ Only one study reported outcomes using disability‐adjusted life years,^12^ whereas another study of school‐based dental check‐up program reported outcomes using prevented‐DMFT, quality‐adjusted tooth years, and per 1% cardholder reached, which is a generic measure for incorporating health inequity.^13^ Using dental‐specific outcome measures for dental interventions or programs do not enable comparability with non‐dental interventions, which is important when considering health investment by policy decision‐makers. Therefore, the present study will expand on this area of knowledge by translating dental health outcomes into a common general health outcome measure: quality‐adjusted life years (QALY).

This paper aimed to perform a cost‐effectiveness analysis of biannual professional application of fluoride varnish in the permanent teeth for an individual aged 15 years and older, compared against the current practice (non‐routine application) from the Australian health care system perspective. Cost‐effectiveness was assessed against the Australian government reference incremental cost‐effectiveness ratio (ICER) threshold of AUD$28 033 per QALY gained.^14^

## 2 METHODS

This study is based on data from previously published literature and publicly available information. Therefore, ethics approval was not required and conducted according to the principles of the Declaration of Helsinki.

### 2.1 Economic evaluation

Two models were used, namely a decision tree and a Markov model. The decision represented the overall mean costs and benefits of both biannual fluoride application (intervention) and current practice (comparator). The Markov model was adapted from a published model that was used to assess the cost‐effectiveness of an oral health intervention.^15^ This Markov model simulates the progression of dental caries of eight permanent molars.^16^ A previous study reported that only 79% of children aged 13 years had all four second permanent molars erupt.^17^ Therefore, the hypothetical cohort aged 15 years was determined at baseline because all eight permanent molars would have fully erupted. The Markov model represented ten mutually possible health states of a single molar that could happen within 6 months (Table 1). The Markov model ran until every individual reached the age of 85 years or died from background mortality.^18^

**Table 1:** The unit cost and utilities associated with the health states

Markov state	Unit cost ($)	DMFT	Utilities
No disease	0	0	1.00
Enamel decay	0	0	1.00
Dentine decay	0	1	0.24
Filling	203	1	0.77
Repeat filling	203	1	0.77
Root canal	883	1	0.77
Crown	1547	1	0.77
Extraction	194	1	0.56
Bridge	2710	1	0.77
Implant	5316	1	0.77

Abbreviation: DMFT, decay, missing and filled teeth.

Different percentages were assigned to each transition state as follows: no decay (82.7%), enamel caries (2.5%), dentine caries (0.3%), first time filled (3.6%), repeated filling (0%), root canal (0%), crown (0%), missing (10.9%), bridge (0%) and dental implant (0%).^16^ Within the 6‐month cycle, a single molar has a chance to move from one health state to another. This chance for moving between health states is known as transitional probabilities, which were adapted from previous studies.^15^, ^16^, ^19^ Consistent with the relevant literature, the incidence of dental caries was assumed to be constant.^16^, ^20^, ^21^, ^22^ Transition probabilities for subjects aged 76‐85 years was extrapolated as for individuals aged 75.

The original Markov model was developed for current practice, and the transition probabilities were based on the second largest Australian private health insurer dental service claims data between 2004 and 2007.^16^ The prevented fraction of biannual application of fluoride varnish was used to adjust relevant transition probabilities for the intervention.

The pooled decayed, missing and filled surfaces (DMFS) prevented fraction was 43% (95% CI 30%, 57%).^3^ Using the prevented fraction, the transition probabilities in the fluoride varnish group moving from “No disease” to “Enamel caries,” and from “Enamel caries” to “Dentine caries” were modified. However, once a molar progressed to the “Filling” health state, it was assumed that fluoride varnish did not have a clinical benefit. The long‐term efficacy of fluoride varnish was assumed to be constant, as was assumed in previous work.^15^, ^16^ The DMFS prevented fraction was converted to the DMFT prevented fraction, which enabled calculations to derive QALY gained.^16^

### 2.2 Costs

Unit costs were based on the 2014 Australian Dental Association fee survey.^16^ No ongoing background costs were assumed for any health state. The cost of the intervention incurred was $37.70 per 6‐month cycle assuming all other resources were the same in the intervention and comparator groups, with different transition probabilities to represent the clinical practice of the two options. While “Repeat filling” can be more expensive than “Filling,” there is no epidemiological evidence to inform what the future costs for “Repeat filling” would be. Therefore, the minimum cost for “Repeat filling” was assumed to be the cost as for “Filling” (the first time the tooth was restored).

### 2.3 Outcomes

The calculated prevented‐DMFT (the DMFT difference between the intervention and comparator) was used to estimate QALY gained by multiplying the utility weight to the number of years stayed in that particular health state. The Australian population utility weights were applied to derived QALY for each molar according to the tooth health state (Table 1).^23^, ^24^ The QALY for an individual was calculated as the average QALY of eight molars.

### 2.4 Discounting

A discounting rate of 5% per annum was applied to both costs and outcomes including 0% and 3.5% discount rates according to the Australian guidelines of the Pharmaceutical Benefits Advisory Committee (PBAC).^25^

### 2.5 Scenario analysis

To assess the robustness of the economic evaluation analysis, we undertook a series of analyses by replacing the mean prevented caries fraction of biannual fluoride varnish with its upper and lower 95th percentile. Three scenarios were considered:

- Scenario 1 Clinical efficacy for fluoride varnish to reduce dental caries from “Dentine caries” and “Fillings”;
- Scenario 2 Clinical efficacy expanded to “Fillings” and “Repeat fillings” in addition to “Dentine caries” and “Fillings”;
- Scenario 3 Costs of two fluoride varnish applications in each 6‐month cycle to quantify the plausible efficacy of quarterly fluoride varnish applications if required.

The model was performed using Microsoft Office Professional Plus 2016 Excel (Microsoft Corporation).

## 3 RESULTS

### 3.1 Base‐case analyses

The results of the base‐case analysis are summarised in Table 2. In total, the model predicted that an individual in the comparator would incur the mean cost of AUD$2303, whereas an individual in the intervention would incur the mean cost of AUD$3600 (95% CI 3483; 3671), inclusive of the AUD$1465 costs for the intervention over the 70‐year time horizon. The individual in the comparator was predicted to have had yielded 15.52 DMFT and 14.74 QALY gained. Compared to current practice, the intervention yielded 13.99 DMFT (95% CI 13.13; 14.57) and 15.44 QALY gained (95% CI 15.19; 15.77).

**Table 2:** The results of the base‐case analysis including the 95% upper and lower limit clinical efficacy of biannual fluoride varnish, discounted

Outcomes	Current practice		Intervention	
	Number	Costs ($)	Number (95% CI)	Costs ($) (95% CI)
Total cost		2303		3600 (1117, 3483)
DMFT	15.52		13.99 (13.13, 14.57)	
QALY gained	14.74		15.44 (15.19, 15.77)	
ICER per prevented‐DMFT			849 (494, 1453)	
ICER per QALY gained			1851 (1142, 3042)	

Abbreviations: CI, confidence interval; DMFT, decay, missing and filled teeth; QALY, quality‐adjusted life years; ICER, incremental cost‐effectiveness ratio.

**Figure 1:**
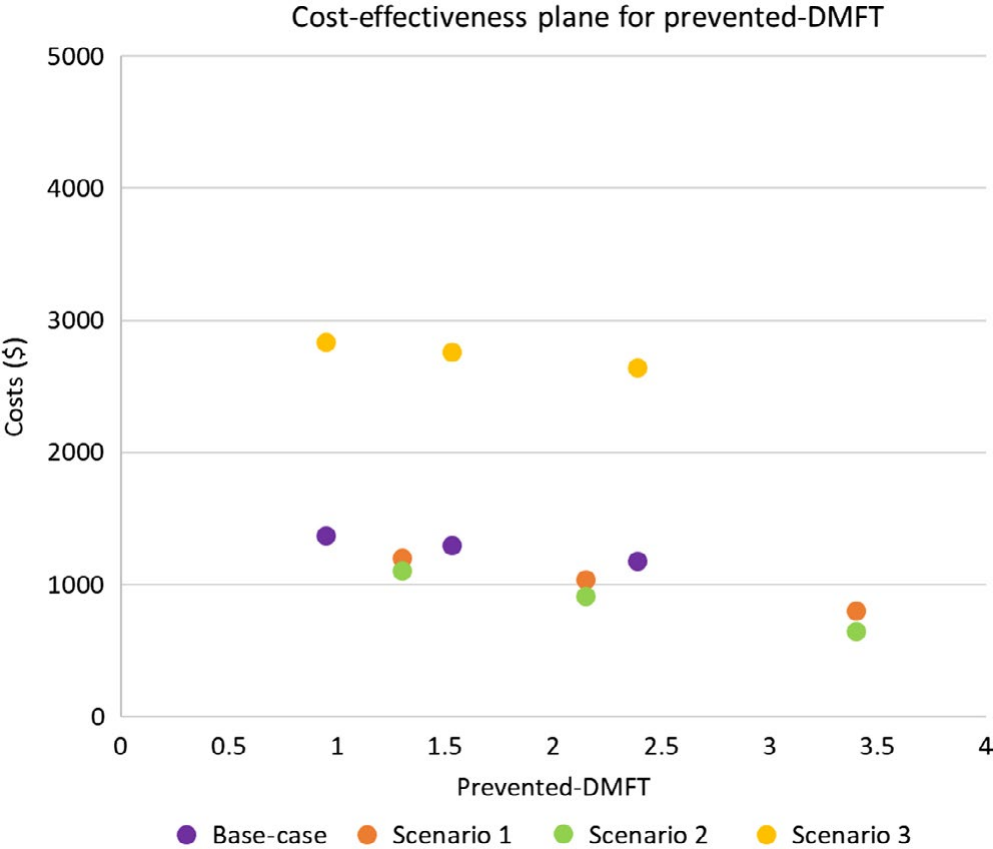
The cost‐effectiveness plan illustrating the incremental cost‐effectiveness ratio values for prevented‐decayed, missing and filled teeth (DMFT), including the 95% confidence intervals

**Figure 2:**
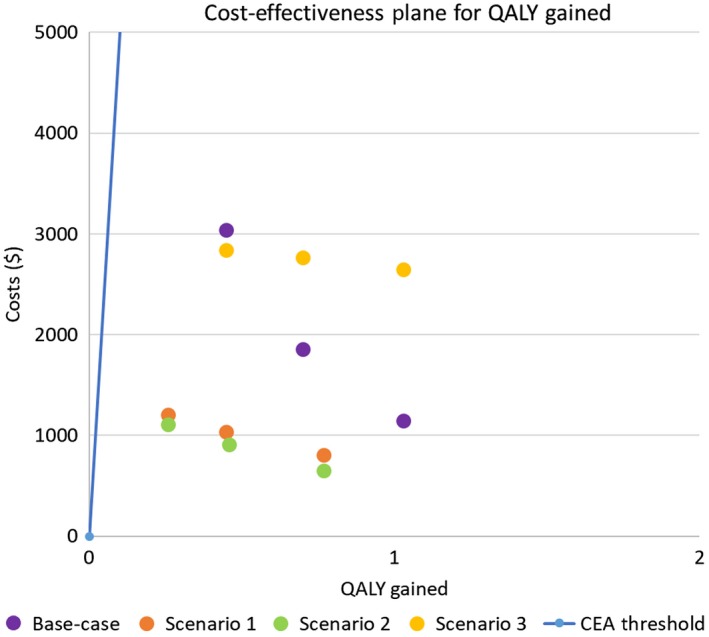
The cost‐effectiveness plan illustrating the incremental cost‐effectiveness ratio values for quality‐adjusted life years (QALY), including the 95% confidence intervals

The intervention was estimated to have an additional cost of AUD$849 (95% CI 494; 1453) per prevented‐DMFT, and AUD$1,851 (95% CI 1142; 3042) per QALY gained, which is below the reference cost‐effectiveness ICER threshold of $28 033 per QALY (Figures 1 and 2). With a 0% annual discount rate, the ICER was AUD$1913 per prevented‐DMFT (95% CI 1117; 3278), and AUD$4354 per QALY gained (95% CI 2727; 7110). With a 3.5% annual discount rate, the ICER was AUD$1009 per prevented‐DMFT (95% CI 591; 1724), and AUD$2231 per QALY gained (95% CI 1389; 3650). Detailed results for the discount rates are presented in Table 3.

**Table 3:** The results for 0% and 3.5% discount rate including the 95% upper and lower limit clinical efficacy of biannual fluoride varnish

Outcomes	0% Discount rate				3.5% Discount rate			
	Current practice		Intervention		Current practice		Intervention	
	Number	Costs ($)	Number	Costs ($)	Number (95% CI)	Costs ($) (95% CI)	Number (95% CI)	Costs ($) (95% CI)
Total Cost		6810		11 472 (11 350, 11 538)		2969		4725 (4602, 4799)
DMFT	59.41		56.97 (55.34, 57.97)		21.49		19.74 (18.72, 20.42)	
QALY gained	47.45		48.52 (48.11, 49.11)		19.25		20.04 (19.75, 20.43)	
ICER per prevented‐DMFT			1913 (1117, 3278)				1009 (591, 1724)	
ICER per QALY gained			4354 (2727, 7110)				2231 (1389, 3650)	

Abbreviations: CI, confidence interval; DMFT, decay, missing and filled teeth; QALY, quality‐adjusted life years; ICER, incremental cost‐effectiveness ratio.

### 3.2 Scenario analyses

The results of the scenario analyses are shown in Table 4. The calculated ICER for each of the scenarios is illustrated in Figure 1 and two on cost‐effectiveness planes. For all scenarios, the intervention was more expensive and more effective.

**Table 4:** Results of the sensitivity analysis for the fluoride varnish arm regarding the three scenarios, discounted

Outcomes	Scenario 1		Scenario 2		Scenario 3	
	Clinical efficacy for “Dentine decay” and “Fillings”		Clinical efficacy as per Scenario 1 and for “Fillings” and “Repeat fillings”		Clinical efficacy as per base‐case scenario with 3‐monthly applications	
	Number (95% CI)	Costs ($) (95% CI)	Number (95% CI)	Costs ($) (95% CI)	Number (95% CI)	Costs ($) (95% CI)
Total cost		3338 (3104, 3503)		3212 (2951, 3410)		5066 (4948, 5137)
DMFT	13.37 (12.12, 14.22)		13.37 (12.12, 14.22)		13.99 (13.13, 14.57)	
QALY gained	15.19 (15.00, 15.51)		15.20 (15.00, 15.51)		15.44 (15.19, 15.77)	
ICER per prevented‐DMFT	482 (235, 923)		424 (190, 852)		1807 (1108, 3010)	
ICER per QALY gained	2287 (1044, 4621)		1999 (843, 4241)		3941 (2561 6299)	

Abbreviations: CI, confidence interval; DMFT, decay, missing and filled teeth; QALY, quality‐adjusted life years; ICER, incremental cost‐effectiveness ratio.

## 4 DISCUSSION

Our findings were consistent with a study that showed biannual fluoride varnish applications are more expensive and more effective than current practice.^21^ Other studies have shown that the intervention is cost‐saving for children under 6 years.^20^, ^26^ A possible reason for cost savings among young children is that the intervention was compared to the costs of subsequent dental treatments that are often performed under general anaesthesia. Our study excluded the potential cost savings from preventable hospitalisation for the treatment of dental caries. Dental conditions are the highest cause of acute potentially preventable hospitalisations among Australians aged under 25 years.^27^

Our study is one of a few Australian‐based economic evaluations of dental caries prevention. Results show that biannual fluoride varnish application is highly cost‐effective compared against the Australian reference ICER threshold of AUD$28 033. Despite dental caries being a significant public health issue, there is complacency about its management and its impact at an individual and societal level. Primary and secondary preventions are not widely applied, whereas surgically biased concepts of dental treatment still predominate within the dental profession.^28^

Biannual fluoride varnish application is not regularly performed in dental practice in Australia. Where it is government funded, the intervention can only be performed by registered dental professionals. This makes access to an effective dental caries prevention method limited for many Australians from priority groups at high risk for dental caries.^29^ Some studies have trained non‐dental professionals to apply fluoride varnish in Australian primary health care settings. Evidence suggests there are clinical benefits for Indigenous children in the Northern Territory.^30^, ^31^, ^32^ Fluoride varnish applications could be performed by non‐dental professionals such as general practitioners, nurse practitioners and midwives, maternal and child health nurses, community pharmacists and Aboriginal and Torres Strait Islander Health Workers.^29^

To address concerns regarding early access to preventive oral health services among children,^33^ there are contemporary discussions in Victoria to expand child population access to fluoride varnish by utilising dental assistants similar to that has been implemented in Scotland for preschool settings.^34^, ^35^ Other settings in which routine biannual fluoride varnish could be applied are within the adult population living in residential aged care facilities, where there is a significant high unmet need for management of dental caries and other oral diseases^.^ 36, 37

However, the application of fluoride varnish by non‐registered health support workers (such as dental assistants) and registered non‐dental professionals need to abide by each state and territory drug and poison regulations. A study to explore the acceptability, knowledge and attitudes of non‐dental professionals of their role in applying fluoride varnish should be conducted. Modelling the cost‐effectiveness of biannual fluoride varnish application by non‐dental professionals was not considered in our study, largely because the costs for professional training to expand their scope of practice in this area are unknown.

This is the first study to perform an economic evaluation of an oral health intervention using “mainstream” health technology assessment methods. Therefore, we were unable to make any comparisons to reports in the current literature. A major strength of the study is that it adds to the limited research on general health outcomes related to oral disease. Our findings also help inform public policy and health investment in oral health. Non‐dental professionals can take an active health promotion role by administering biannual fluoride varnish applications, which are non‐invasive, cost‐effective and relatively easy to apply in non‐dental settings.

### 4.1 Limitations

There are several limitations to our study. First, the benefit of dental caries prevention on non‐molar teeth was omitted from our analysis. The cost‐effectiveness results are likely to underestimate the total clinical benefit of biannual fluoride varnish applications.

Second, the transition probabilities were based on the data from a private health insurer, which were drawn from a population who are likely to have better oral health than the general population. Furthermore, the intervention only captured the costs and transition probabilities of eight molar teeth from a total of 28 teeth in a standard human dentition.

Third, the same transition probabilities were applied to every age cohort for individual teeth, and therefore, dental caries risk. There are likely variations in dental caries risk factors among different age groups, which would vary the transition probabilities. Furthermore, the transition probabilities were derived from dental records of people who had private health insurance cover, which inherit the “affordability” confounder. The transition probabilities may not represent those from lower socio‐economic backgrounds, who are likely to have higher dental caries risk and untreated dental caries. Since the current transitional probabilities did not include untreated dental caries, the ICER is likely to be underestimated.

Fourth, we assumed that biannual fluoride varnish was applied with 100% adherence throughout the entire time horizon. In reality, adherence would be less than 100%, meaning efficacy would be less than ideal. The lower levels of adherence would also lead to lower costs of the intervention. Hence the impact on cost‐effectiveness would be more or less balanced.

Fifth, the use of a constant discount rate can be a significant limitation, in which its application may not truly represent the values of a society.^38^ However, the Australian PBAC guidelines currently do not adopt a declining discount rate as the principal approach in economic evaluations.^25^

Finally, the estimation of QALY gained from prevented‐DMFT was subject to uncertainty. Due to very little advancement in economic evaluations in dentistry, not all ten states in the model had available utility weights, which required us to make assumptions on these utilities in our model. Our analysis conservatively presumed the utility weights for enamel decay, root canal, crown, bridge and implant had the same utility as filling because the tooth was filled and symptom‐free.

## 5 CONCLUSION

Biannual application of fluoride varnish in Australian children aged 15 years over a 70‐year horizon is likely to be highly cost‐effective. The intervention should be adopted in routine dental practice and perhaps supported more broadly within primary health care settings provided by non‐dental professionals. Biannual fluoride varnish applications should be considered for universal funding in Australia's health care system.

## **CONFLICT OF INTEREST**S

Susan Cartwright is employed by the Colgate‐Palmolive Company. She was not involved in the study design, data collection, data analysis and does not have access to the study data. She was only involved in the initial conception of the study design, revising the paper critically for important intellectual content and final approval of the paper.
